# Referrals from community optometrists to the hospital eye service in Scotland and England

**DOI:** 10.1038/s41433-021-01728-2

**Published:** 2021-08-06

**Authors:** Rakhee Shah, David F. Edgar, Abeeda Khatoon, Angharad Hobby, Zahra Jessa, Robert Yammouni, Peter Campbell, Kiki Soteri, Amaad Beg, Steven Harsum, Rajesh Aggarwal, Bruce J. W. Evans

**Affiliations:** 1 Centre for Applied Vision Research, City, University of London, London, UK; 2 Glasgow Caledonian University, Glasgow, UK; 3 Cardiff University, Cardiff, UK; 4 RNIB, London, UK; 5 Institute of Optometry, London, UK; 6 University of Plymouth, Plymouth, UK; 7 Specsavers Opticians, St. Andrew, Guernsey; 8 Norwich Medical School, Norwich, UK; 9 Epsom and St Helier University Hospitals NHS Trust, Epsom, UK; 10 Southend University Hospital, Southend-on-Sea, UK

**Keywords:** Health services, Health occupations

## Abstract

**Objectives:**

This audit assesses communication between community optometrists (COs) and hospital eye service (HES) in Scotland and England.

**Methods:**

Optometric referrals and replies were extracted from six practices in Scotland and England. If no reply was found, replies/records were copied from HES records. De-identified referrals, replies and records were audited against established standards, evaluating whether referrals were necessary, accurate and directed to the appropriate professional. The referral rate (RR) and referral reply rate (RRR) were calculated.

**Results:**

From 905 de-identified referrals, RR ranged from 2.6 to 8.7%. From COs’ perspective, the proportion of referrals for which they received replies ranged from 37 to 84% (Scotland) and 26 to 49% (England). A total of 88–96% of referrals (Scotland) and 63–76% (England) were seen in the HES. Adjusting for cases when it is reasonable to expect replies, RRR becomes 45–92% (Scotland) and 38–62% (England) with RRR significantly greater in Scotland (*P* = 0.015). Replies were copied to patients in 0–21% of cases. Referrals were to the appropriate service and judged necessary in ≥90% of cases in both jurisdictions. Accuracy of referral ranged from 89 to 97% (Scotland) and 81 to 98% (England). The reply addressed the reason for referral in 94–100% of cases (Scotland) and 93–97% (England) and was meaningful in 95–100% (Scotland) and 94–99% (England).

**Conclusions:**

Despite the interdisciplinary joint statement on sharing patient information, this audit highlights variable standard of referrals and deficits in replies to the referring COs, with one exception in Scotland. Replies from HES to COs are important for patient care, benefitting patients and clinicians and minimising unnecessary HES appointments.

## Introduction

Community optometrists (COs) perform most primary eyecare consultations in the United Kingdom (UK). In England, the eye examination is either privately funded by the patient or funded by the NHS (70% of sight tests) through the General Ophthalmic Services sight test (GOS-ST) [1]. In Scotland, everyone is entitled to an NHS-funded community eye examination. In 2018–2019, 13.2 million NHS-funded eye examinations [2] were conducted in England and 2.3 million in Scotland [3].

The primary purpose of the GOS-ST in England is to detect, measure and correct refractive error. The GOS-ST in England includes a basic ocular health check to determine whether the patient needs a referral to their general practitioner (GP) or hospital eye service (HES). In Scotland, subsequent to the extended General Ophthalmic Services (GOS) legislation (2010), the NHS eye examination not only provides a primary eyecare service, enabling optometrists to detect early signs of problems, but also allows optometrists to manage these in partnership with the HES. Eye examinations carried out by COs in Scotland are either primary (performed at established intervals depending on the patient’s age and ocular health) or supplementary (performed outside and in addition to these established intervals).

Most new patients attending the HES originate from optometric referrals [4]. Up-to-date information on the proportion of optometric eye examinations that result in referrals is lacking. Previous studies of optometric referrals have evaluated those patients who reach the HES [4–11], though these studies do not provide data on what proportion of optometric referrals attend an appointment in the HES. NHS Scotland reported that the referral rate (RR) from community optometry to HES increased from 2.5% in 2010/2011 to 4.1% in 2018/2019 [12]. A recent analysis of approximately 650,000 GOS-ST forms in England reveals an overall RR of 5.1%, with patients aged 60 years and above being four times more likely to be referred than children [13]. These previous referral studies [4–13] concentrated on initiation of referrals. There is a dearth of studies investigating optometric referrals from their initiation through to the HES appointment and any consequent correspondence.

The quality of referrals [6, 10, 14–16] may contribute to, or be adversely affected by, the reported rarity of replies to the referring optometrist [17]. Lack of feedback to COs could impact on public health (discussed below). The Royal College of Ophthalmologists and College of Optometrists published a joint statement on sharing of patient information in 2015 [18], noting that ‘Optometrists, as regulated professionals, are part of the healthcare team so it is usually in the patients’ best interest for ophthalmologists to share clinical information with the referring optometrist’. They concluded that hospitals should send copies of GP letters to the referring optometrist unless the hospital policy specifically prohibits this. Subsequently the Primary Care Division of the Scottish Government’s Population Health Improvement Directorate issued two memorandums reinforcing the statement, noting that ‘It is also expected that meaningful feedback will be provided to optometrists, copied to the patients GP, if a patient is seen by the ophthalmologist’ [19, 20]. The Caldicott review [21] advocates sharing of information between healthcare professionals.

The overarching aims of this audit are to assess optometric referrals and replies, identify differences in RRs and referral reply rates (RRRs) between Scotland and England and investigate whether these referrals conform to standards in the College of Optometrists Guidance for Professional Practice [22, 23], the joint statement and subsequent memorandums [18–20] and the Caldicott review [21]. The audit investigates the appropriateness, necessity and accuracy of optometric referrals to the HES, whether made via the GP or directly, the proportion for which the optometrist receives a reply and how often replies are copied to the patient. The audit also asks whether replies address the reason for referral and are meaningful; and quantifies the RR, the proportion of referrals that reach the HES and the RRR.

## Methods

The UK Health Research Authority (HRA) confirmed that the study is an audit not requiring HRA review. Approval from local R&D Departments was obtained as appropriate.

Following a lengthy search and consideration of approximately 15 potential pairings (dyads) of CO practices and HES clinics in different areas of England and Scotland, three suitable dyads were identified in Scotland and compared with three dyads in England. Key requirements for participating dyads are detailed elsewhere [24]. In summary, typical CO practices and HES units were sought that would be supportive of the audit and did not have unusually strong links between the practice and HES.

### Phase 1: CO practice data extraction

Audit team members initially visited each CO practice (2–5 days) extracting de-identified copies of referral letters and replies. The initial audit period of 18 months, ending 6 months before the start of the audit, was extended back in time or shortened until 150 referrals or 100 replies were obtained, whichever was reached sooner. Information on the total number of GOS-STs and private eye examinations completed in each practice during the audit period was used to calculate the RR for each practice.

### Phase 2: HES data extraction

The audit team visited each HES unit, extracting de-identified copies of correspondence relating to referrals for which no reply was found in the dyad CO practice. For referred patients who had attended the HES, but no report written, de-identified copies of relevant records were obtained [24].

### Phase 3: data analysis

A senior audit team member (dyad co-ordinator) entered key data from de-identified copies of referrals and replies in a spreadsheet [24]. Data that required clinical judgement (outcomes 1–3 in Table 1 and additional information items 4a and 4b) were subsequently graded by the audit team. The spreadsheet and de-identified referral and reply letters were securely shared with the audit team, together with any relevant information about the dyad (e.g., special referral pathways for cataract, AMD, glaucoma, etc.).

**Table 1:** Summary of key outcomes and standards used in the audit.

	Audit outcome	Standard
1	Is the referral to an appropriate professional? (a) from the referrer’s perspective (b) from an overall perspective	C.Optom guideline C152: [21] referrals should be ‘to a practitioner with the appropriate knowledge & skills’
2	Is the referral necessary?	C.Optom guideline C143: [21] refer ‘a sign or symptom of injury or disease which you cannot manage’
3	Is the referral accurate?	GOC rules (1999): [39] referral should be written report ‘indicating grounds for thinking the person may be suffering from injury or disease of the eye’
4	What proportion of optometric referrals receive a reply? (referral reply rate; RRR)	Joint statement: [17] ‘ophthalmologists should send copies of GP letters to the referring optometrist’ Memorandum from Scottish Government: [18] ‘meaningful feedback will be provided to optometrists, copied to the patient’s GP, if a patient is seen by the ophthalmology department’
5	Of optometric referrals that result in a letter to the GP and/or optometrist, for what proportion does the patient receive a copy?	Caldicott review: [20] ‘all communications between different health and social care teams should be copied to the patient’

*C.Optom* College of Optometrists, *GOC* General Optical Council, *GP* general (medical) practitioner.

All clinical judgements and gradings were made independently by the senior audit team (BE, DE, RS, ZJ) after reviewing the referral letters and replies, with every sixth case independently graded by a member of a multi-disciplinary expert panel, comprising two COs with considerable experience in community practice (in the independent and corporate sectors), hospital optometry and optometric education and training.

Each outcome or additional information item was graded, using the guidelines described below, answering as yes, no, N/A or unknown. Graders noted any ‘uncertain’ gradings and discussed these, and cross-checked gradings where there was disagreement, at meetings of members of the senior audit team and expert panel. Meeting attendees reviewed referrals and replies, reaching agreement by consensus.

### Outcomes

Audit outcomes and the standards with which audit results were compared are summarised in Table 1. Some outcomes refer to College of Optometrists Guidance for Professional Practice, using the edition (2014) relevant to the audit [22].

Outcome 1, ‘Is the referral to an appropriate professional?’, was considered first from the referrer’s perspective, solely taking account of information in their referral letter. Second, after viewing any reply or the hospital record, the question was reconsidered from the overall perspective, including HES findings. To standardise the criteria for interpreting outcomes 1–3 (Table 1), guidelines were operationalised for 14 commonly referred conditions [24]. For each condition, common scenarios were listed and mapped to outcomes 1–3, following review from the expert panel.

Additional information was gathered as follows:

1. Proportion of NHS eye examinations in Scotland and GOS-STs and private eye examinations in England resulting in referral (RR).
2. Proportion of optometric referrals whose ultimate destination was the HES and the proportion to other services (e.g., private ophthalmology; GP).
3. Proportion of optometric referrals directed to HES that attend HES.
4. Whether replies to referrals (a) address the reason for referral and (b) are meaningful.

All key proportions are calculated as percentages, followed by the 95% confidence interval (binomial ‘exact’ method). Where *χ*^2^ tests have been carried out, *P* values below 0.05 are taken as statistically significant.

### Calculation of referral reply rate (RRR)

To calculate RRR, the number of referrals to the audit HES unit must be calculated. This involves deducting from all referrals: duplicate referrals, referrals to GP not intended for HES; private referrals, referrals to other HES units and referrals not to HES (e.g., to neurologists). Two different methods of calculating the RRR were applied.

Apparent RRR (aRRR) is the number of replies in COs’ records from any HES unit divided by the number of referrals to HES and is the only estimate available to studies without access to HES data. To calculate the modified RRR (mRRR), the numerator is the number of replies from the audit HES unit in COs’ records. The denominator only includes patients seen in the audit HES unit after deducting: any replies found in HES and clearly addressed or copied to the optometrist (though no copy was found in the COs practice), referrals not requiring a response (e.g., letter of information) and replies sent after the date of the audit team visit to the COs practice. The number deducted for these reasons was very low.

## Results

### Demographics

Of the three Scotland dyads (S1, S2, S3), two were in the central belt and the third in northeast Scotland. One practice is an independent, one part of a small corporate chain and one from a corporate chain of >150 practices. The three comparison England dyads (E1, E2, E3) are in the East of England, Outer London and the South of England. In England, two practices are independents and one from a corporate chain of >150 practices. The Office of National Statistics database was used to determine the 2018 gross disposable household income per head (GDHI) for the local authorities where dyads are located [25].

The format of referral letters varied. For both Scotland and England, referral letters in two venues were all typed and in the third all handwritten. Audit periods, demographics and RR are summarised in Table 2.

**Table 2:** Audit periods and age (years) demographics of optometric referrals in the six practices.

Practice	Audit period	Mean age	Median age	Min. age	Max. age	GDHI (£)	RR (%) (95% confidence interval)
S1	Aug 2016 to July 2017	64	70	3	94	19,000	7.2 (6.1–8.4)
S2	Aug 2016 to Sept 2017	61	68	1	94	19,500	2.6 (2.2–3.0)
S3	June 2016 to Aug 2017	68	72	1	90	21,500	7.0 (4.3–10.7)
E1	Mar 2016 to Jan 2018	66	71	4	93	24,500	3.6 (3.1–4.2)
E2	Aug 2015 to Oct 2017	64	69	4	92	18,500	8.7 (6.7–11.0)
E3	May 2015 to Nov 2017	66	72	3	93	28,000	6.5 (5.5–7.6)

*Min* minimum, *Max* maximum, *GDHI* gross domestic household income per head, *£* pounds sterling, *RR* referral rate.

### Numerical data

A total of 905 de-identified referral letters were extracted from six CO practices. Table 3 shows the key numerical calculations and data. aRRR varied from 36.8% (28.7–45.5%) to 83.7% (76.0–89.8%) for Scotland and from 25.5% (18.6–33.6%) to 48.8% (39.9–57.8%) for England. aRRR differed significantly in the three dyads (*χ*^2^ test, *P* < 0.001) for both England and Scotland. mRRR varied from 44.6% (35.2–54.3%) to 92.0% (85.3–96.3%) for Scotland and 38.1% (27.7–49.3%) to 61.8% (50.0–72.8%) for England, and differences between dyads were significant both in Scotland (*P* < 0.001) and in England (*P* = 0.005). When all the data in Scotland were pooled and compared with the pooled data from England, there was a significant difference between the two jurisdictions for both aRRR (*χ*^2^ test, *P* < 0.001) and mRRR (*P* = 0.015).

**Table 3:** Overview of data and calculation of referral reply rate.

		Number at each dyad					
	Variable	Dyad S1	Dyad S2	Dyad S3	Dyad E1	Dyad E2	Dyad E3
a	Total referrals extracted	154	150	137	157	152	150
b	Duplicates	1	1	0	2	3	0
c	Private referrals	0	7	2	3	1	11
d	Referrals to HES not requiring reply (letter of info)	0	0	0	1	0	0
e	Referrals to GP, not for onward referral	15	5	7	10	6	12
f	Other non-ophthalmology referrals	2	1	5	0	1	0
g	Referrals intended for HES when reply appropriate (=a–b–c–d–e–f)	136	136	123	141	141	127
h	Replies from any HES unit found in CO practice	57	50	103	36	59	62
i	**Apparent referral reply rate: aRRR** (=h/g)	**41.9% (33.5–50.7%)**	**36.8% (28.7–45.5%)**	**83.7% (76.0–89.8%)**	**25.5% (18.6–33.6%)**	**41.8% (33.6–50.4%)**	**48.8% (39.9–57.8%)**
j	Referrals directed to audited HES unit(s)	136	136	123	118	140	120
k	Referrals to an unaudited HES unit	0	0	0	23^a^	1	7
l	Records sought in HES (=g–h)	79	86	20	105	82	65
m	Records found in HES	71	84	20	52	48	32
n	Report sent to GP but not to CO practice	58	73	9	50	44	32
o	Reply sent to CO, but not found in audit visit	7	11	4	0	3	0
p	Record in audit HES unit, but not attended by date of audit	8	11	5	0	0	0
q	Reply sent to CO after audit visit	0	0	2	0	0	0
r	Total number known to be seen in HES (=h+m–p)	120	123	118	88	107	94
s	Patients who had appointments at HES unit in audit	120	123	118	84	106	76
t	% of patients referred to audit HES unit, who attended (=s/j)	88.2	90.4	95.9	71.2	75.7	63.3
u	Replies in CO records from audit HES unit	57	50	103	32	59	47
v	Patients seen at HES audit unit where reply apposite (=s–o–q)	113	112	112	84	103	76
w	**mRRR** (=u/v)	**50.4% (40.9–60%)**	**44.6% (35.2–54.3%)**	**92.0% (85.3–96.3%)**	**38.1% (27.7–49.3%)**	**57.3% (47.2–67.0%)**	**61.8% (50.0–72.8%)**
**x**	**% of patients who are sent a copy of the referral reply**	**0**	**6.7 (3.1–12.2%)**	**1.3 (0.20–5.8%)**	**6.0 (2.0–13.4%)**	**2.8 (0.6–8.0%)**	**20.7 (12.9–30.4%)**

Key audit outcomes are highlighted in bold, with 95% confidence intervals in parentheses.

*HES* hospital eye service, *GP* general (medical) practitioner, *CO* community optometrist, *aRRR* apparent referral reply rate (see text), *mRRR* modified referral reply rate (see text).

^a^At this dyad, there were other proximal HES units and some patients were referred there.

Of 7 (Scotland) and 15 (England) private referrals across the three CO practices, 100% (Scotland) and 73.3% (44.9–92.2%) in England generated a reply to the referring optometrist. Few referrals solely intended for GPs’ attention received a reply: 3/27 in Scotland and 3/28 in England.

### Content of referral letters and replies

The referral was to the appropriate professional (Table 1, outcomes 1a and 1b) in at least 90% of cases for Scotland and >95% for England and the referral was considered necessary in >90% of cases for both Scotland and England (Table 4). The referral was considered accurate in >89% of cases in Scotland and over 80% in England. The reply addressed the reason for referral in 94–100% of cases (Scotland) and 93–97% (England) and was meaningful in 95–100% (Scotland) and 94–99% (England).

**Table 4:** Summary of gradings of referral letters and replies.

Audit outcome number		% at each dyad					
↓	Audit outcome description	Dyad S1	Dyad S2	Dyad S3	Dyad E1	Dyad E2	Dyad E3
1a	Is the referral to an appropriate professional – referrer’s perspective?	97.4 (93.4–99.3)	99.3 (96.3–100)	100.0 (97.1–100)	100.0 (97.6–100)	99.3 (96.3–100)	99.3 (96.3–100)
1b	Is the referral to an appropriate professional – overall perspective?	90.0 (83.2–94.7)	99.2 (95.4–100)	99.1 (95.3–100)	100.0 (95.4–100)	95.6 (89.0–98.8)	97.8 (92.2–99.7)
2	Is the referral necessary?	90.8 (84.2–95.3)	97.5 (92.9–99.5)	96.6 (91.4–99.1)	96.3 (89.7–99.2)	92.9 (86.0–97.1)	96.7 (90.6–99.3)
3	Is the referral accurate?	89.2 (82.2–94.1)	96.6 (91.5–99.1)	97.4 (92.6–99.5)	97.5 (91.3–99.7)	81.1 (71.7–88.4)	94.4 (87.4–98.2)
Addit.	Do replies address the reason for referral?	94.2 (88.4–97.6)	97.5 (92.8–99.5)	100.0 (96.9–100)	92.9 (85.3–97.4)	96.4 (89.9–99.3)	96.7 (90.6–99.3)
Addit.	Are replies meaningful?	95.0 (89.4–98.1)	98.3 (94.1–99.8)	100.0 (96.9–100)	94.0 (86.5–98.0)	96.4 (89.9–99.3)	98.9 (94.0–100)

*Addit.* additional information.

## Discussion

### Comparison of dyads

Although six dyads cannot be fully representative of referrals in the jurisdictions, they spanned large areas of both countries, contained rural and urban environments and had a range of socio-economic profiles and GDHIs in areas covered by the dyads. The target of 150 referrals or 100 replies from each practice was met in every case.

The aRRR, indicating COs’ perspective of the RRR, ranged from 37 to 84% in Scotland compared with 26–49% in England. For the English dyads (particularly the dyad on the outskirts of London), the proximity of alternative HES units to the practices resulted in some referrals being directed to, or subsequently choosing to attend, an unaudited HES unit (row k, Table 3). This is thought to be an infrequent occurrence but may have accounted for some of the 30% of referrals to the audit HES unit in England who did not attend that unit. The dyads associated with the lowest household income (S1 and E2) had the highest RR. This may be linked to the poor uptake of GOS-STs by deprived communities [26], and could contribute to the association between deprivation and sight-threatening conditions [27].

The mRRR, the proportion of replies for referrals known to reach the HES where a reply is appropriate, varies from 38% in E1 to 92% in S3. Interestingly, despite greater NHS use of COs and better integration with the HES in Scotland, the mRRRs for two Scottish dyads (50.4% and 44.6% in S1 and S2, respectively) were within the range of the three mRRRs in England (38.1–61.8%). A clear outlier, in terms of communication between ophthalmology and optometry is dyad S3, which overwhelmingly meets the standards set out in the joint statement [18] and memorandums from the Scottish Government [19, 20], and highlights what can be achieved. A number of factors contributed to the outstanding performance of this Scottish dyad, but the overarching factor is the quality of two-way communication between COs and the HES. Communication links include: a central direct telephone link for COs to seek advice from a member of the nursing team or, if necessary, from a duty ophthalmologist, with an option to book the patient into an urgent-access clinic; an email advice line; direct referral from the CO to the HES via secure NHS mail; and regular training for COs from ophthalmologists at evening meetings. Some or all of these measures may be in place elsewhere but in this exemplar dyad the measures are used regularly and have fostered excellent inter-professional relationships and communication. When all data for Scotland were pooled and compared with pooled data for England, both the mRRR and aRRR were significantly higher in Scotland.

The body of the Caldicott review ‘recommends that all communications between different health and social care teams should be copied to the patient or service user’ [21]. Our audit identifies between 0 and 7% in Scotland and 3 and 21% in England of cases where a copy of the reply was intended for the patient. Although these figures are approximate due to limited audit time in the optometric practices, it is likely that correspondence is infrequently copied to patients/primary carers. There is no formal recommendation on this issue in the Caldicott review report.

The Scottish arm of this audit adds to the evidence base concerning the proportion of patients seen in the HES following referral by COs, with approximately 10% of those referred in Scotland not attending an appointment at the HES compared with one third in England (Table 3, row t).

### Comparison with previous work

COs have been criticised for over-referring [28], which may, in part, explain why optometrists often do not receive a reply to referrals they make to the HES [17]. Another factor may be the quality of COs’ referrals, which has received criticism [6, 10, 15–17]. Many optometrists now refer directly to the HES, making it particularly surprising that although the optometrist often receives no reply, the GP receives a reply to a referral they were not involved in. This could in part be explained by the historical design of HES patient record systems.

Previous studies of communication between COs and HES units have only considered either referrals from the CO practice [29], or information found in the HES [4–11, 14, 16, 30]. Uniquely, our audit investigates communication from the perspective of both primary and secondary care, resulting in two RRRs. From the COs’ perspective, the aRRR is simply the proportion of referral letters that receive a reply. The mRRR is from the HES perspective, taking account of those referrals that do not attend the HES and some that do not require a reply. In Scotland, the mRRR was approximately 8% higher than the aRRR, compared with a 12–15% difference in England.

Optometric referrals are the main source of patients seen in the HES; however, little is known about the proportion of community eye examinations resulting in referral (the RR). Previous studies reveal an overall RR of 3.6–5.1% [1, 13]. In the present work, the RR (2.6–8.7%) varied considerably between practices, with similar differences between the England and Scotland dyads investigated. This variation is unlikely to be explained by patient age, since the mean, median and range of age of patients referred are similar in all six practices. There was no significant correlation between GDHI and RR for the six practices (rho = −0.60, *P* = 0.21), although the England data alone indicate that the RR could reflect a higher proportion of pathology in low income areas [31]. Regarding the proportion of referrals seen in the HES, attendance rates of 82 and 70% have been reported for glaucoma referrals from CO practices [32, 33]. Our figures range from 63 to 96% overall, with a statistically significant (*χ*^2^, *P* < 0.0001) difference between Scotland (88–96%) and England (63–76%).

Research into optometric continuous professional development (CPD) reveals no significant effect of CPD on referral management decisions [34]. Generic advice via CPD lacks the relevance of real cases, and the likely best approach to raising optometric referral standards is for referrals to receive replies. This creates a feedback loop, allowing the optometrist to consider the ophthalmologist’s opinion when deciding on referring a patient with a similar clinical presentation.

To our knowledge, this study is the first systematic approach to calculating RRR. Although one dyad in Scotland had an impressively high aRRR (84%) and mRRR (92%), this dyad was an outlier from the other five dyads with aRRRs from 26 to 49% and mRRRs from 38 to 62%. Clearly, for some practices most optometric referrals do not receive a reply, impacting future referral quality.

In the absence of a reply from the HES, COs often must ask patients, when they reattend the practice, for the outcome of the HES appointment. Patients may not accurately recall the HES findings. Without useful information on the HES outcome an unnecessary re-referral may result, adding to NHS costs, and wasting an HES appointment slot at a time when sight loss is occurring whilst patients are on HES waiting lists [35]. Copying the referral reply to the referring optometrist is likely to minimise unnecessary re-referrals, reducing patient anxiety and sight loss.

### Strengths and limitations

Strengths of this work include the large sample size of 900+ referrals in Scotland and England, and the bilateral approach to investigating the referral pathway. By obtaining de-identified copies of referral letters/replies, we assessed their exact content. The retrospective design prevented practitioners altering referral behaviour, as might occur in a prospective study. Inevitably, grading referrals/replies for quality required subjective judgement, although measures were taken to minimise subjectivity [24].

A limitation is that the England dyads were in the southern half of England, although one was north of Birmingham. Optometrists in all regions of England follow the same NHS contract regarding community sight tests, must comply with General Optical Council standards and typically follow College of Optometrists guidelines including those relating to referrals. Therefore, at the time of the research it is unlikely that there were significant systematic variations in optometric referrals in different regions of England [36].

The results of this audit reveal considerable variations in performance between dyads. This variation, which is notable, together with the small number of dyads in each country reduces the generalisability of the outcomes of comparisons between Scotland and England.

Poorly performing practitioners may be less likely to participate in studies of this type, while clinics with good relationships between CO practice and HES may be more likely to participate. Dyads were sought where the CO practice primarily referred to one HES unit. Such settings are probably more likely to have a good relationship than settings where a CO refers to several HES units. As a result of both these limitations, this audit is likely to have over-estimated the quality of communication between COs and the HES.

## Conclusions

The audit finds an overall high but somewhat variable standard of optometric referrals in England and Scotland, and highlights an overall deficit in replies, although the reply rate varies amongst HES units, with an impressively high reply rate in one exemplar dyad in Scotland. Referral replies help maintain high standards of patient care, avoid unnecessary re-referral and close the feedback loop, thereby raising the standard of referrals. Correspondence from the HES in response to COs’ referrals is only infrequently copied to the patient or their primary carer(s). This has important implications post COVID-19 where COs are carrying out some functions previously undertaken in the HES [37, 38]. The next generation of online referral platforms alongside new ophthalmology electronic patient record systems should be designed to ensure that summary information from HES consultations is routinely accessible to both the referring optometrist and GP.

### Summary

#### What was known before

- In the UK, most new patients seen in the hospital eye service originate from community optometrist (CO) referrals.
- Reported rarity of replies to the referring optometrist means the CO cannot determine whether the patient was seen and whether the problem for which they were referred has been addressed.

#### What this study adds

- Despite the interdisciplinary joint statement on sharing patient information, this audit highlights variable standards of referrals and deficits in replies to the referring COs.
